# ChSte7 Is Required for Vegetative Growth and Various Plant Infection Processes in* Colletotrichum higginsianum*

**DOI:** 10.1155/2016/7496569

**Published:** 2016-08-03

**Authors:** Qinfeng Yuan, Meijuan Chen, Yaqin Yan, Qiongnan Gu, Junbin Huang, Lu Zheng

**Affiliations:** The Key Laboratory of Plant Pathology of Hubei Province, Huazhong Agricultural University, Wuhan, Hubei 430070, China

## Abstract

*Colletotrichum higginsianum* is an important hemibiotrophic phytopathogen that causes crucifer anthracnose in various regions of the world. In many plant-pathogenic fungi, the Ste11-Ste7-Fus3/Kss1 kinase pathway is essential to pathogenicity and various plant infection processes. To date, the role of ChSte7 in* C*.* higginsianum* encoding a MEK orthologue of Ste7 in* Saccharomyces cerevisiae* has not been elucidated. In this report, we investigated the function of ChSte7 in the pathogen. The ChSte7 is predicted to encode a 522-amino-acid protein with a S_TKc conserved domain that shares 44% identity with Ste7 in* S*.* cerevisiae*. ChSte7 disruption mutants showed white colonies with irregularly shaped edges and extremely decreased growth rates and biomass productions. The ChSte7 disruption mutants did not form appressoria and showed defects in pathogenicity on leaves of* Arabidopsis thaliana*. When inoculated onto wounded leaf tissues, the ChSte7 disruption mutants grew only on the surface of host tissues but failed to cause lesions beyond the wound site. In contrast, both the wild-type and complementation strains showed normal morphology, produced appressoria, and caused necrosis on leaves of* Arabidopsis*. Analysis with qRT-PCR suggested that ChSte7 was highly expressed during the late stages of infection. Taken together, our results demonstrate that ChSte7 is involved in regulation of vegetative growth, appressorial formation of* C*.* higginsianum*, and postinvasive growth in host tissues.

## 1. Introduction

The hemibiotrophic ascomycete fungus* Colletotrichum higginsianum* is the causal agent of anthracnose disease on a wide range of cruciferous plants, such as* Brassica*,* Raphanus*, and the model plant* Arabidopsis thaliana* [1]. For example, in South China, this fungus usually causes typical water-soaked lesions on leaves of Chinese cabbage (*B*.* parachinensis*), leading to 30–40% yield loss yearly [2]. To invade host tissue, conidia first attach to plant surfaces and germinate to form melanized appressoria. After that,* C*.* higginsianum* penetrates the plant cell with high turgor pressure generated in the melanized appressorium, and then large bulbous biotrophic hyphae form in the first infected cell. Finally, the fungus differentiates secondary hyphae to kill host tissues [3].

The* C*.* higginsianum*-*A*.* thaliana* pathosystem provides an attractive model for dissecting fungal pathogenicity and plant resistance, in which both partners can be genetically manipulated [4]. Genome and transcriptome analyses of* C*.* higginsianum* infecting* A*.* thaliana* indicate that this fungus has many virulence factors [5]. To date, just limited molecular determinants of virulence in* C*.* higginsianum* have been reported. ChEC effectors are focally secreted from appressorial penetration pores before host invasion, revealing new levels of functional complexity for* C*.* higginsianum* [6]. Arginine biosynthesis was shown to be critical for early stages of plant infection by* C*.* higginsianum* [7]. Ch-MEL1 is required for both appressorial formation and melanin production in* C*.* higginsianum* as well as postinvasive growth in host tissues [8]. Chpma2 deletion mutants form fully melanized appressoria but entirely fail to penetrate the host tissue and are nonpathogenic [9]. However, more virulence factors in the model phytopathogen remain to be elucidated and characterized.

Signal transduction is a highly conserved system that enables eukaryotes to sense and respond to extracellular conditions. The mitogen-activated protein kinase (MAPK) cascade is one of the ubiquitous signaling systems in eukaryotes. The cascade is universally composed of three kinase proteins, MAPK-extracellular regulated kinase kinase (MEKK), MAPK-extracellular regulated kinase (MEK), and MAPK [10]. Upon perception of an appropriate external stimulus, MEKK phosphorylates MEK, which then phosphorylates MAPK, resulting in enzymatic activation and eventual relay of signal to ultimately activate physiological responses [11, 12]. These pathways are involved in a variety of developmental processes in yeasts and filamentous fungi [12, 13]. The signaling model in yeast,* Saccharomyces cerevisiae* involving the Ste11-Ste7-Fus3/Kss1 cascade, has been characterized for pheromone responses and filamentous growth pathways [14]. In several phytopathogenic filamentous fungi, MAPK genes have been frequently annotated as virulence factors, such as Pmk1 in* Magnaporthe oryzae* [15–17], Cmk1 in* C*.* orbicular* [18], Kpp2 (Ubc3) in* Ustilago maydis* [19, 20], and Bmp1 in* Botrytis cinerea* [21]. The results of previous studies indicate that there are some differences in this signaling system between fungal plant pathogens and yeasts. In* S*.* cerevisiae*, certain protein kinases act in more than one pathway (e.g., the MEK Ste7 and MEKK Ste11 participate in two and three pathways, resp.); however, the Ste11-Ste7-Kss1 cascade is unique to pathogenesis in plant-pathogenic fungi [12, 13]. With the exception of differences in signaling systems, it is suggested that there are significant differences in the input/output of this cascade, not only between yeasts and plant pathogens, but also among different pathogens [15, 18–20]. The inputs and outputs of these cascades are probably dependent on the fungal species, which suggests that the components of the MAPK cascades should be separately characterized for individual fungal plant pathogens.

The Ste11-type MEKK and Ste7-type MEK are two important upstream kinases of the Fus3/Kss1-type MAPK cascade which has been considered a master regulator of pathogenesis in plant pathogens [22, 23]. In previous work, the Ste7 homologues in many pathogens were found to be essential for appressorial formation in pathogenesis [24–28]. In this study, we identified and characterized ChSte7 encoding a MAPKK orthologue of the yeast Ste7 in* C*.* higginsianum*. Deletion of ChSte7 resulted in significant reduction in vegetative growth and loss of ability to form appressoria. Most interestingly, wounding inoculation assays and microscopic observations indicated that the ChSte7 deletion mutants were also defective in their invasive growth inside the host plant tissues. Otherwise, ChSte7 was highly expressed in the postinvasive growth phase. All these data support the involvement of ChSte7 in regulation of vegetative growth, appressorial formation, and postinvasive growth in host tissues.

## 2. Materials and Methods

### 2.1. Strains, Plasmids, and Plants

The wild-type strain IMI349061 of* C*.* higginsianum* (Table 1), originating from diseased tissues of* B*.* campestris*, was kindly provided by Professor Yangdou Wei from the University of Saskatchewan, Canada. Plasmids pMD18T-HYG with hph cassette and pNeo3300III with neocassette used for gene disruption and complementation vector construction [29] were stored at −80°C in 20% glycerol (v/v) as bacterial suspensions.

*Arabidopsis thaliana* ecotype Col-0 was used in virulence assays.* Arabidopsis* seeds were sown on the surface of peat-based compost and placed in growth chamber with 16/8 h photoperiod and day and night temperatures of 22 and 18°C, severally. Lighting provided a photosynthetic photon flux rate of 40 *μ*mol m^−2^s^−1^ (400–700 nm), and the chamber was maintained at 65–80% relative humidity.

### 2.2. Disruption and Complementation of Target Gene ChSte7

To replace ChSte7, an 870 bp fragment of upstream flanking sequence and 950 bp fragment of downstream flanking sequence of the gene were amplified, respectively, with primer pairs, ChSte7F1FP/ChSte7F1RP and ChSte7F2FP/ChSte7F2RP (Table 2). PCR products of the upstream flanking sequence digested with HindIII/SalI and the downstream flanking sequence digested with XbaI/KpnI were ligated into the corresponding restriction sites of vector pMD18T-HYG, resulting in the initial vector F1-HYG-F2. The vector F1-HYG-F2 was then digested with HindIII and KpnI and ligated with pNeo3300III to form the gene disruption vector pNeo3300IIIChSte7-Ko. The vector, pNeo3300IIIChSte7-Ko, was transformed into* Agrobacterium tumefaciens* EHA105 by electroporation, and then conidia of* C*.* higginsianum* wild-type strain were transformed with vector pNeo3300IIIChSte7-Ko based on the* A*.* tumefaciens*-mediated transformation (ATMT) protocol described by Li et al. [30]. To obtain ChSte7 disruption mutants, transformants were grown on potato dextrose agar (PDA) supplemented with 50 *μ*g/mL of hygromycin (Merck, Germany) and 500 *μ*g/mL of cephalosporin (Amresco, USA) and then subcultured on PDA supplemented with 150 *μ*g/mL antibiotic G418 (Amresco, USA). Gene disruption transformants were confirmed by PCR amplification with two pairs of primers, ChSte7-KF/ChSte7-KR and HphSP/HphAP (Table 2), and RT-PCR with primers ChSte7-KF and ChSte7-KR (Table 2).

To confirm that phenotypes of the ChSte7 disruption mutants were due to the targeted gene disruption, one disruption mutant* ΔChSte7-26* (Table 1) was complemented with a full length sequence of ChSte7. Since the upstream sequence of ChSte7 was not found in sequencing scaffolds of the* C*.* higginsianum* assembly, high-efficiency thermal asymmetric interlaced PCR (hiTAIL-PCR) was used to amplify the upstream sequence of ChSte7 (corresponding to the promoter region). For hiTAIL-PCR, genomic DNA was used as a template in successive reactions with nested RB-specific primers RB-0a, RB-1a, and RB-2a together with the degenerate primers LAD1-1, LAD1-2, LAD1-3, LAD1-4, and AC1 following thermal cycling settings for hiTAIL-PCR described by Liu and Chen [31]. The 3882 bp fragment including the 1728 bp ORF of ChSte7, 1674 bp upstream, and 480 bp downstream was amplified from genomic DNA of the wild-type strain with primer pair Ste7comFP and Ste7comRP (Table 2) and cloned into the HindIII site of vector pCAMBIA3300III, generating the complementation plasmid pNeo3300IIIChSte7-Com. To obtain the ChSte7 complementation transformants, conidia of Δ*ChSte7-26* were transformed with vector pNeo3300IIIChSte7-Com by the ATMT method. The complementation transformants were screened on PDA containing 150 *μ*g/mL G418, and gene fragments were detected by PCR and RT-PCR analyses.

### 2.3. Phenotypic Analysis

Mycelia of the wild-type strain and mutants were inoculated onto PDA plates and cultured in darkness for 7 days at 25°C for growth rate and conidiation testing. Mycelia were harvested by suction filtration from 7-day-old cultures grown in 100 mL potato dextrose broth (PDB) at 25°C with shaking at 150 rpm, dried at 60°C, and weighed. Hyphae picked from edge of the colony on PDA were examined by light microscopy (Nikon, Tokyo, Japan). Conidia were harvested with sterile distilled water and passed through four layers of lens paper to remove debris and mycelia. The conidial suspension was adjusted to a concentration of 1 × 10^6^ spores/mL with sterile deionized water and suspension droplets (10 *μ*L) were spotted on microscope plastic coverslips (Thermo Fisher Scientific, MA, USA) placed in 9 cm diameter petri dishes, and conidial germination and appressorial formation were examined by light microscopy after 12 h.

### 2.4. Pathogenicity Assay and Infection Observation

Conidial suspensions at the concentration of 1 × 10^6^ spores/mL were prepared as stated above and used for plant inoculation. Intact plants of* Arabidopsis* were used to assess the virulence of the disruption and complementation transformants of ChSte7. Conidial suspensions were sprayed onto the upper and lower surfaces of* Arabidopsis* leaves from 4- to 5-week-old plants. After sealing the plants inside plastic propagators lined with wet tissue paper to provide high humidity, inoculated plants were incubated at 25°C in a controlled environment chamber (18 h photoperiod). Lesion formation was examined at 5 days after inoculation (dpi).

To observe infection structures of wild-type strain and mutants,* Arabidopsis* leaves from 4- to 5-week-old plants were spotted with 10 *μ*L droplets of the prepared conidial suspension on either side of each midvein. Inoculated leaves were incubated in complete darkness at 25°C. Inoculated leaf tissues collected after 4 days of incubation were cleared in a solution of methanol : chloroform : glacial acetic acid (6 : 3 : 1), then rehydrated and stained with 1% trypan blue in glycerol, and viewed by light microscopy.

To assess the ability of the ChSte7 disruption mutants to grow invasively inside the host plant tissues independent of penetration, conidial suspensions were also spotted on wounded site of the* Arabidopsis* leaves. Wounding experiments were carried out by pricking the detached leaves with a fine sterile needle prior to inoculation and placing conidial suspensions directly on the wound sites. Lesion formation was examined at 4 dpi and inoculated leaf tissues were cleared, rehydrated and stained, and finally viewed by light microscopy.

### 2.5. DNA/RNA Manipulation, RT-PCR, and qRT-PCR Analysis

Total genomic DNA (gDNA) was isolated from* C*.* higginsianum* wild-type strain with CTAB following Sambrook et al. [32]. Hyphae harvested from PDB, conidia obtained from PDA plates, and* Arabidopsis* leaves sprayed with conidial suspension at concentration of 1 × 10^6^ spores/mL at 5, 20, 40, 65, and 90 h after incubation (hpi) were collected, flash-frozen in liquid nitrogen, and stored at −80°C until required. RNA isolation was carried out using TRIzol® Plus RNA Purification Kit (Invitrogen, Carlsbad, USA), and potential DNA contamination was removed by DNase I treatment (RNase Free) (Takara, Dalian, China) following manufacturer's instructions. First-strand cDNA was synthesized by using Revert Aid first-strand cDNA synthesis kit (Fermentas, St. Leon-Rot, Germany) following the manufacturer's instructions. Expressions of ChSte7 in disruption mutants and the complementation strain were examined by RT-PCR, and a 1208 bp fragment was amplified with gene-specific primers ChSte7-KF and ChSte7-KR (Table 2). The* C*.* higginsianum β*-tubulin sequence amplified with primers TubulinS and TubulinA (Table 2) was used as the reference gene. PCR conditions were 25 cycles of 94°C for 30 s, 55°C for 30 s, and 72°C for 1 min and with a final extension at 72°C for 5 min. Expression of ChSte7 at different development stages of the fungus* in vitro* or* in planta* was analyzed by qRT-PCR with ChSte7 gene-specific primers qRT-STE7F/qRT-STE7R (Table 2). The* C*.* higginsianum β*-tubulin as the reference gene was amplified with primers qRT-tubulinF and qRT-tubulinR (Table 2). PCR conditions were 50 cycles of 95°C for 15 s, 55°C for 20 s, and 72°C for 20 s and with a final extension from 65°C to 95°C (0.5°C/5 s). PCR reactions were run on a PTC-200 DNA Engine Peltier Thermal Cycler (Bio-Rad, Hercules, USA).

### 2.6. Bioinformatics

The full sequence of ChSte7 was downloaded from the* C*.* higginsianum* genome database (http://www.broadinstitute.org/annotation/genome/colletotrichum_group/MultiHome.html). To confirm sequence validity, the primers ChSte7FP and ChSte7 RP (Table 2) were designed and used for the amplification of the ChSte7 gene. All primers used in this work were designed with primer premier 5.0 (http://www.premierbiosoft.com/primerdesign/). Open reading frames were further analyzed using the gene prediction program FGENESH (Softberry Inc., Mount Kisco, NY, USA). Protein domain and motif predictions were performed with SMART software (http://smart.embl-heidelberg.de/). The Ste7 protein sequences from different organisms were obtained from the GenBank database, using the BLAST algorithm. Sequence alignments were performed using the Clustal X (version 2.0, http://www.clustal.org/clustal2/), and the phylogenetic tree was generated by the Mega software (version 5.0, http://www.megasoftware.net/index.php).

### 2.7. Statistical Analysis

The data of all quantitative assays were analyzed with DPS statistical analysis software (version 3.01, China Agric. Press, Beijing, China), using analysis of variance and the test of Least Significant Difference (LSD) at *P* = 0.05.

## 3. Results

### 3.1. Identification and Characterization of ChSte7

**Figure 1:**
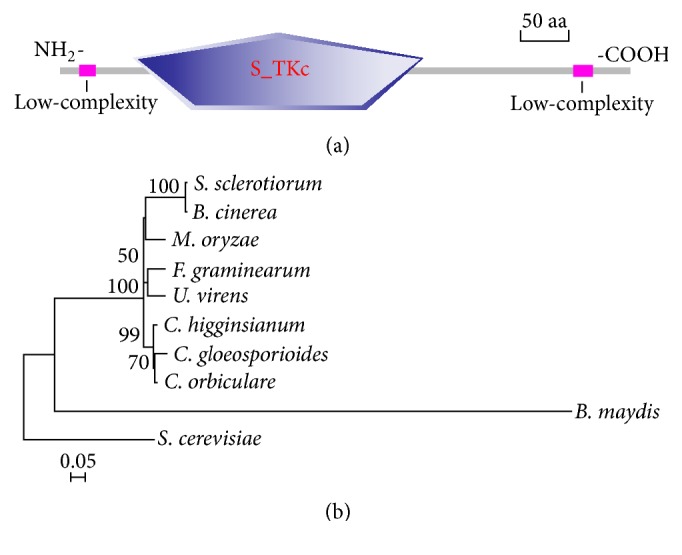
Functional domain identification and phylogenetic tree. (a) A conserved S_TKc domain (serine/threonine protein kinases, catalytic domain) and two low-complexity regions in ChSte7 were predicted using the SMART website. (b) Sequence alignments were performed using Clustal X 2.0 program and the calculated phylogenetic tree was viewed using Mega 5.0 program. Neighbor-joining tree was constructed with 1000 bootstrap replicates of phylogenetic relationships between Ste7 homologues in fungi. All 10 protein sequences of the Ste7 homologues were downloaded from the NCBI database and the accession numbers of Ste7 homologues are shown as follows:* S*.* sclerotiorum* (*Sclerotinia sclerotiorum* XP_001588345.1),* B*.* cinerea* (*Botrytis cinerea* XP_001557712.1),* M*.* oryzae* (*Magnaporthe oryzae* ELQ32975.1),* F*.* graminearum* (*Fusarium graminearum* XP_011318809.1),* U*.* virens* (*Ustilaginoidea virens* KDB12657.1),* C*.* higginsianum* (*Colletotrichum higginsianum* CCF40893.1),* C*.* gloeosporioides* (*Colletotrichum gloeosporioides* AAD55385.1),* C*.* orbiculare* (*Colletotrichum orbiculare* ENH81835.1),* B*.* maydis* (*Bipolaris maydis* EMD86379.1), and* S*.* cerevisiae* (*Saccharomyces cerevisiae* CAA98732.1).

The protein sequence of Ste7 from* S*.* cerevisiae* was used as the query to blast (BLASTp) against available* C*.* higginsianum* genome. We identified a locus CH063_02455 named as ChSte7 which encodes the Ste7 homologue and is predicted to encode a 522-amino-acid protein that shares 44% identity with Ste7 in* S*.* cerevisiae*. Sequence analysis with SMART revealed that ChSte7 contained a S_TKc conserved domain (serine/threonine protein kinases, catalytic domain) (Figure 1(a)). Phylogenetic analysis of ChSte7 to other Ste7 proteins revealed that ChSte7 from* C*.* higginsianum* was most similar to Ste7 proteins of* C*.* gloeosporioides* and* C*.* orbiculare* and most distant from those of* Bipolaris maydis* and* S*.* cerevisiae* (with identities still close to or above 44%) (Figure 1(b)). This result indicates that Ste7 proteins are conserved in fungi.

### 3.2. ChSte7 Is Highly Expressed during Invasive Growth

**Figure 2:**
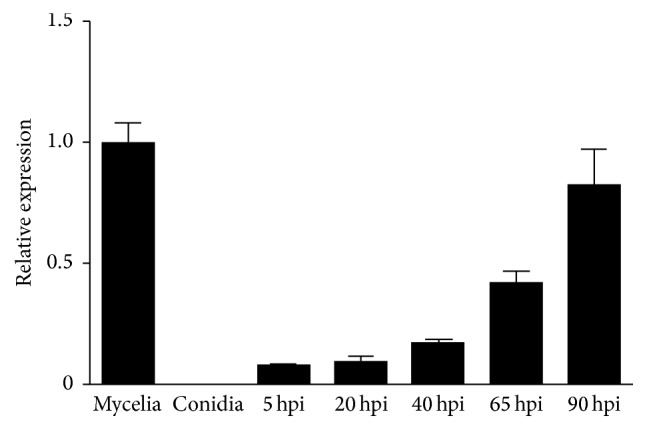
Expression profiles of ChSte7 assayed by qRT-PCR. RNA was extracted from mycelia and conidia, as well as infected* Arabidopsis* seedlings at different times of inoculation (5, 20, 40, 65, and 90 hpi). *β*-tubulin gene was used as an internal control. Relative abundance of ChSte7 transcripts during infectious growth was normalized by comparing with vegetative growth in potato dextrose broth (relative transcript level = 1). Three independent biological experiments with three replicates in each treatment were performed.

To gain insight into the functions of ChSte7, we first examined the gene expression profile at different stages of* C*.* higginsianum* using qRT-PCR. In comparison to the conidiation stage, the expression levels of ChSte7 were significantly increased in vegetative and invasive growth stages (5 to 90 hpi) (Figure 2). The expression of ChSte7 was highest in the vegetative stage while no expression was found in the conidiation stage. Lower expressions were detected during the early stages of infection at 5–40 hpi. However, the expression of ChSte7 increased significantly during late infection until 90 hpi. These observations suggest that ChSte7 is highly expressed in the late infection and may play a key role during necrotroph in* C*.* higginsianum*.

### 3.3. Targeted Disruption and Complementation of ChSte7

**Figure 3:**
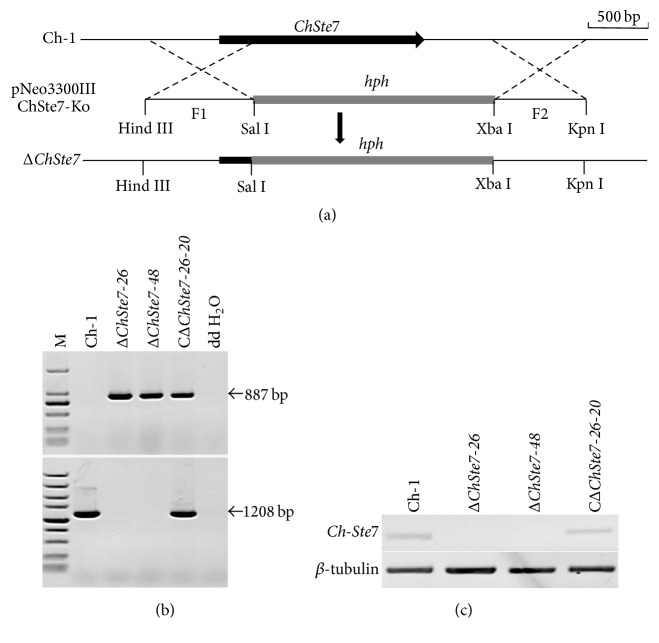
Targeted disruption and complementation of ChSte7 gene. (a) ChSte7 locus and gene deletion vector. (b) PCR analysis of wild-type strain (Ch-1), ChSte7 disruption mutants (Δ*ChSte7-26* and Δ*ChSte7-48*), and complementation strain (CΔ*ChSte7-26-20*). Markers (M) in the top and bottom images are DL2000 and DL5000, respectively. The band of 877 bp in hph gene was amplified in the ChSte7 disruption mutants and complementation strain while a 1208 bp fragment in ChSte7 gene was obtained in the wild-type and complementation strains. (c) RT-PCR analysis of wild-type strain, ChSte7 disruption mutants, and complementation strain.

A gene disruption vector, pNeo3300IIIChSte7-Ko (Figure 3(a)), containing hygromycin B phosphotransferase (hph) gene and both 5′ and 3′ flanking regions of ChSte7, was constructed and then transformed into the wild-type strain. The transformants were first selected on hygromycin-containing media and then selected on G418-containing media to avoid random insertion. Fifty candidate disruption transformants without resistance to G418 among 348 hygromycin-resistant transformants were obtained. Two candidate disruption transformants Δ*ChSte7-26* and Δ*ChSte7-48* were found lacking the 1208 bp ChSte7 fragment compared to the wild-type strain after PCR amplification with STE7SP/STE7AP (Table 2), but an 887-bp hph fragment was obtained by PCR amplification with hphF/hphR (Table 2) in the two candidate transformants (Figure 3(b)). Null mutation of the ChSte7 gene was further confirmed by RT-PCR analysis, since the ChSte7 transcript was not detected in these two targeted disruption transformants (Figure 3(c)). These results demonstrated that the ChSte7 gene was deleted in the ChSte7 disruption transformants Δ*ChSte7-26* and Δ*ChSte7-48*. To investigate whether altered growth phenotypes and the loss of virulence in ChSte7 disruption transformants could be restored by reintroduction of a wild-type copy of ChSte7, we transformed Δ*ChSte7-26* with plasmid pNeo3300IIIChSte7-Com. Subsequently, complementation transformant CΔ*ChSte7-26-20* was confirmed by PCR and RT-PCR analysis (Figures 3(b) and 3(c)) and chosen for further phenotype analysis.

### 3.4. ChSte7 Plays a Crucial Role in Vegetative Growth and Colony Morphology

**Figure 4:**
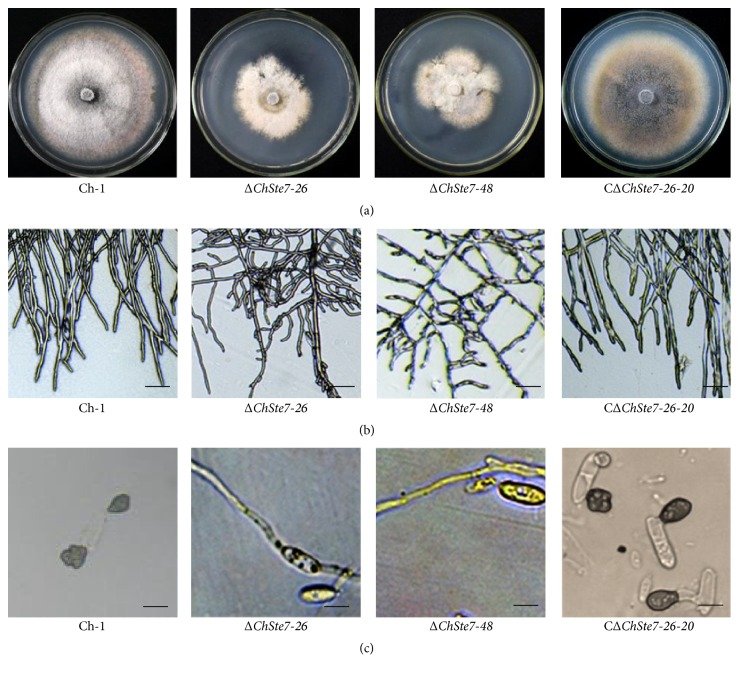
Mycelial growth and appressorial formation of ChSte7 disruption mutants and complementation strain. (a) The ChSte7 disruption caused abnormal colony. Wild-type strain Ch-1, ChSte7 disruption mutants Δ*ChSte7-26* and Δ*ChSte7-48*, and complementation strain CΔ*ChSte7-26-20* were grown on PDA plates for 7 days. (b) Increased furcation of mycelial tips and twisted hyphae were observed for the Δ*ChSte7* mutants. Hyphae of Ch-1, ChSte7 disruption mutants, and complementation strain picked from the edge of the colonies were examined by light microscopy. Scale bar = 10 *μ*m. (c) Appressorial formation was altered in the ChSte7 disruption mutants. Conidial suspensions of Ch-1, ChSte7 disruption mutants, and complementation strain in distilled water were incubated on hydrophobic surface at 25°C for 24 h. Scale bar = 5 *μ*m.

To explore the role of ChSte7 in vegetative growth and colony morphology, the Δ*ChSte7* mutants and the complementation transformant together with the wild-type strain were cultured on PDA plates for 7 days. The Δ*ChSte7* mutants showed a significantly reduced growth rate and biomass while conidial production was consistent with the wild-type and complementation strain (Table 3). The growth rate and biomass of the Δ*ChSte7* mutants were reduced to approximately 55.8–58.1% and 31.6–38.8%, respectively, compared with those of the wild-type strain. The Δ*ChSte7* mutants showed irregular-shape colonies, as well as increased aerial hyphae (Figure 4(a)). Moreover, the Δ*ChSte7* mutants produced wavy and twisted hyphae with increased branching (Figure 4(b)). These results suggest that ChSte7 plays an important role in hyphal growth and morphology.

### 3.5. ChSte7 Is Essential for Appressorial Formation and Pathogenicity in* C*.* higginsianum*

In order to investigate whether ChSte7 is essential for pathogenicity, we first tested the ability to form appressoria by placing droplets of conidial suspensions on artificial hydrophobic surface. The wild-type strain started to form many appressoria by 12 h after inoculation. In contrast, the Δ*ChSte7* mutants germinated poorly (Table 3) and did not form any appressoria, even after 24 h (Figure 4(c)). Moreover, Δ*ChSte7* mutants often formed long germ tubes. The ChSte7 complementation strain CΔ*ChSte7-26-20* regained the ability to form normal appressoria (Figure 4(c)).

**Figure 5:**
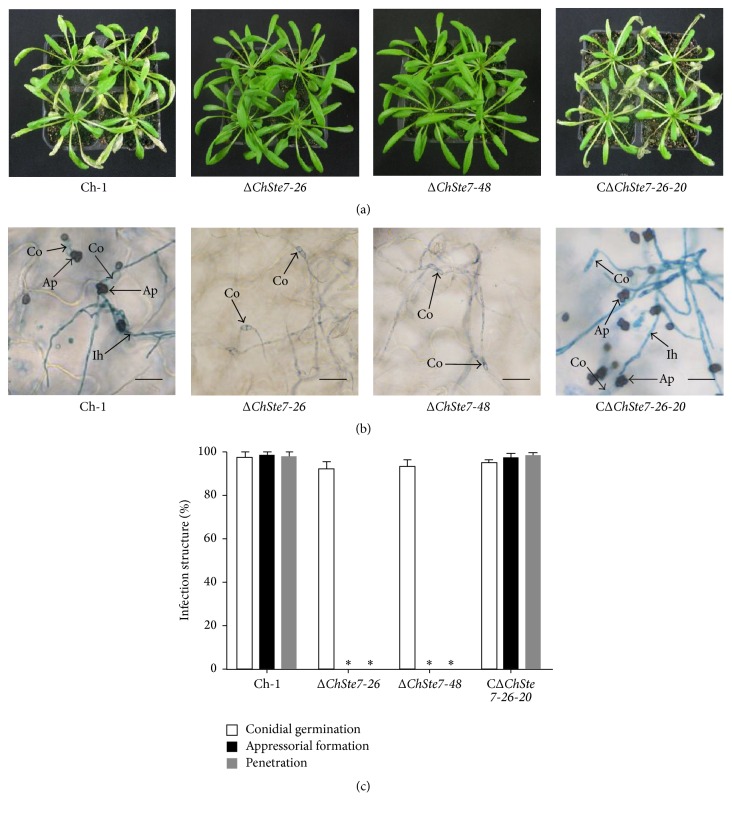
Deficiency in pathogenicity and appressorial formation of ChSte7 disruption mutants on leaves of* Arabidopsis* plants. (a) The ChSte7 disruption attenuated pathogenicity on* Arabidopsis* leaves. Conidia suspensions of wild-type strain Ch-1, ChSte7 disruption mutants Δ*ChSte7-26* and Δ*ChSte7-48*, and complementation strain CΔ*ChSte7-26-20* were sprayed onto the* Arabidopsis* leaves incubated at 25°C for 5 days. (b) Conidial suspensions of wild-type strain, ChSte7 disruption mutants, and complementation strain were inoculated onto the surfaces of* Arabidopsis* leaves and incubated for 4 days. The appressoria of the ChSte7 disruption mutants were not formed, whereas the appressoria of CΔ*ChSte7-26-20* and Ch-1 penetrated epidermal cells and produced abundant invasive hyphae. Ap, appressoria; Co, conidia; Ih, invasive hyphae. Scale bar = 20 *μ*m. (c) Development of infection structures by wild-type strain, ChSte7 disruption mutants, and complementation strain on* Arabidopsis* leaves at 4 dpi. Ratings for infection structures were given as a percentage of the preceding structure. In each experiment, at least 300 appressoria per strain were examined. Means and standard deviations were calculated from three repeated experiments. Asterisks indicate values significantly different from the wild-type (LSD, *P* < 0.05), and standard error bars are shown.

To test the pathogenicity of mutants, conidial suspensions of all strains were inoculated onto nonwounded* Arabidopsis* leaves. The Δ*ChSte7* mutants were nonpathogenic while the wild-type and CΔ*ChSte7-26-20* strain were virulent and formed typical necrotic lesions on leaves (Figure 5(a)).

To verify whether the loss of virulence of the Δ*ChSte7* mutants was attributable to the defect in appressorial formation, we investigated the formation of different infection structures by Δ*ChSte7* mutants on* Arabidopsis* leaves. At 4 dpi, high frequencies of appressorial formation and penetration on the surface of* Arabidopsis* leaves were found in both the wild-type and CΔ*ChSte7-26-20* strain (above 90%; Figures 5(b) and 5(c)). The Δ*ChSte7* mutants germinated but did not form appressoria on the plant surface, and also no invasive hyphae were observed in the epidermal cells (Figures 5(b) and 5(c)), indicating that loss of pathogenicity of the Δ*ChSte7* mutants was caused, at least in part, by defects in appressorial formation. These observations showed that ChSte7 is required in the early stage of the infectious process, which corresponds with the results of appressorial formation experiments.

### 3.6. ChSte7 Disruption Mutants Are Avirulent on Wounded Leaf Tissues

**Figure 6:**
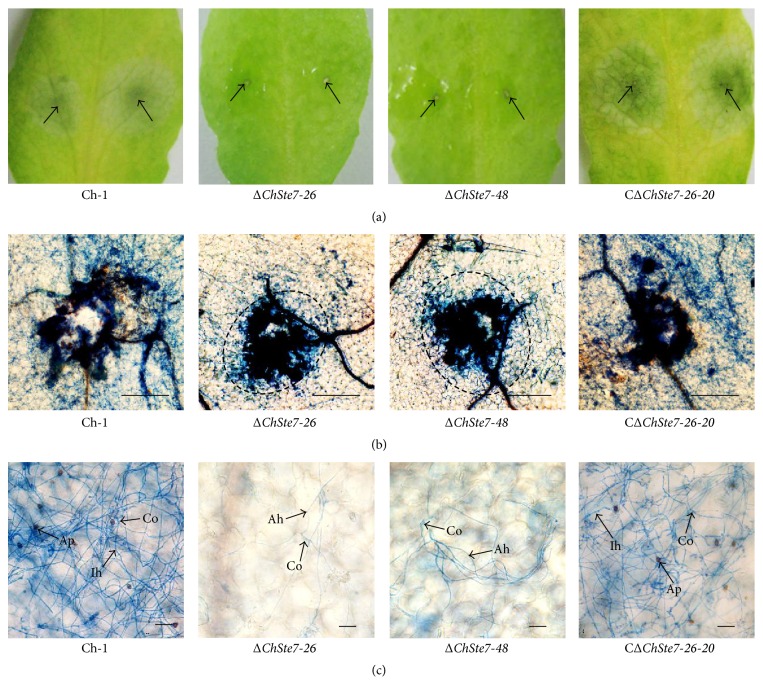
Loss of the ability for invasive growth of ChSte7 disruption mutants in leaves of* Arabidopsis*. (a) Pathogenicity assays were performed on wounded leaves of* Arabidopsis* plants using conidia suspensions of wild-type strain Ch-1, ChSte7 disruption mutants Δ*ChSte7-26* and Δ*ChSte7-48*, and complementation strain CΔ*ChSte7-26-20.* At 4 dpi, the ChSte7 disruption mutants caused no symptoms on wounded tissues, while wild-type and complementation strain caused water-soaked lesions on wounded leaf tissues. Arrows indicate wounded sites on leaves. (b) Leaf tissues from wound sites were viewed by light microscopy. Hyphae from germinating conidia of the ChSte7 disruption mutants grew above the epithelial cells of* Arabidopsis* leaves and could not enter into the wound sites. Scale bar = 100 *μ*m. (c) Near the wound sites, appressoria of the ChSte7 disruption mutants were not formed on leaves, and germinating conidia could not form invasive hyphae to enter into the wound sites but produce aerial hyphae beyond or around wound sites. Ah, aerial hyphae; Ap, appressoria; Co, conidia; Ih, invasive hyphae. Scale bar = 10 *μ*m.

To assess the ability of the Δ*ChSte7* mutants to grow invasively inside the host plant tissues independent of penetration, conidia of the Δ*ChSte7* mutants were directly inoculated on the wound sites of* Arabidopsis* leaves. At 4 dpi, the Δ*ChSte7* mutants could not form lesions on wounded leaf tissues while the wild-type and the CΔ*ChSte7-26-20* strain caused dark necrotic lesions on inoculation sites (Figure 6(a)). Microscopic observation showed that conidia of the Δ*ChSte7* mutants never formed appressoria on* Arabidopsis* leaves, and germinating conidia could not form invasive hyphae to enter into the wound sites but did produce aerial hyphae beyond or around wound sites (Figures 6(b) and 6(c)). The results indicated that Δ*ChSte7* mutants lost the ability for invasive growth in the host tissues. Therefore, we concluded that the loss of virulence in Δ*ChSte7* mutants was attributable to the defects in both appressorial formation and invasive growth.

## 4. Discussion

The MAPK cascade is one of the most important signaling systems in the regulation of morphogenesis and stress responses. Most filamentous fungi including* C*.* higginsianum* possess three conserved MAPK cascades. One of the cascades, the Ste11-Ste7-Fus3/Kss1-type cascade, has been considered as a master regulator of pathogenesis in plant pathogens [13, 22, 23, 33]. The similar phenotypes of mutants in this cascade were observed in previous studies for* M*.* oryzae* [15, 16, 27, 28],* B*.* cinerea* [21, 34],* C*.* lagenarium* [18, 35],* U*.* maydis* [19, 20], and other plant pathogens. These findings implied that Ste7-type MEK was an essential component of the Ste11-Ste7-Fus3/Kss1-type cascade. The Fus3/Kss1-type cascade has been well characterized in some model species of fungal plant pathogens; however, two upstream kinases, Ste11-type MEKK and Ste7-type MEK, have only been characterized in a few systems. In this study, we identified and characterized Ste7-type MEK in* C*.* higginsianum*, in order to elucidate the functional roles of Ste7, using disruption mutant strains. Consequently, we found Ste7-type MEK in* C*.* higginsianum* to be involved in various stages of development and morphogenesis in lifecycles, such as hyphae development, appressorial formation, and postinvasive growth. This is the first description of roles of Ste7-type MEK in the model phytopathogen* C*.* higginsianum.*

Homologues of Ste7 MEK are involved in appressorial formation, an important infection-related morphogenetic process, in* M*.* oryzae* [27, 28],* Colletotrichum* spp. [18, 25, 35],* B*.* cinerea* [21, 34], and* U*.* maydis* [19, 20, 26] as well as* B*.* maydis* [24, 36, 37]. We suggest that the Fus3/Kss1-type MAPK cascade has highly conserved roles in appressorial formation among plant pathogens. If the involvement of ChSte7 was confined to the initial penetration into the host, then the disruption mutant should be able to infect a host with a mechanically breached outer surface. However, ChSte7 disruption mutants of* C*.* higginsianum* were unable to form lesions on wounded* Arabidopsis* leaves. Moreover, ChSte7 was highly expressed in the late infection but not in early infection stage. We suggest that Ste7-type MEK might be a crucial factor in pathogenicity of other necrotrophic fungi.

In this study, the results showed that the Ste7-type MEK was not involved in conidiation but did affect conidial germination in* C*.* higginsianum*. The ChSte7 disruption mutants produced as many conidia as the wild-type strain, but the conidia of ChSte7 disruption mutants germinated poorly (6%) on plastic coverslips. However, the germination rate of conidia of ChSte7 disruption mutants was more than 92% at 5 dpi on* Arabidopsis* leaves. We speculate that the inhibition of conidial germination in ChSte7 disruption mutants might be overcome on leaf surfaces by host signals and nutrients. Previous studies indicated that the Fus3/Kss1-type MAPK cascade regulated conidiation in* M*.* oryzae* [15, 27] and other* Colletotrichum* spp. [18, 25, 35] as well as* B*.* maydis* [25, 36, 37]. On the other hand, the level of regulation of conidial germination was significantly different among these species; no conidia germinated in* B*.* maydis* [25, 36, 37], a few conidia germinated in* M*.* oryzae* [15, 27], and a few conidia germinated in water but most conidia of* Colletotrichum* spp. germinated in nutrient-rich conditions [18, 25, 36]. In contrast, this cascade of* B*.* cinerea* seemed not to be involved in conidiation but affected conidial germination in pure water [21]. In* U*.* maydis*, both formation and germination of teliospores were also regulated by this cascade [19, 20, 26]. Therefore, the Ste11-Ste7-Fus3/Kss1-type MAPK cascade could regulate morphogenesis of sexual or asexual spores in the species mentioned above, although the regulated stage, spore formation or germination, differed depending on the species.

In* S*.* cerevisiae*, Ste7 MEK is essential for signal transduction from Ste11 MEKK to Kss1/Fus3 MAPK [12, 38, 39]. However, Ste11 MEKK also acts upstream of another MAPK cascade, the Hog1-type MAPK cascade. In this pathway, called the “Sho1 branch,” Ste11 MEKK transmits the signal to downstream proteins via Pbs2 MEK, not Ste7 MEK [40, 41]. On the other hand, previous studies indicated that this crosstalk of MAPK cascades should not occur in filamentous fungi, including plant pathogens, because Pbs2-type MEK in filamentous fungi lacks the proline-rich motif that is required for binding to the Sho1 sensor protein [42–44]. Hence, it is suggested that Ste7-type MEK may transmit all signals from Ste11-type MEKK to Fus3/Kss1 MAPK, without any crosstalk with another MAPK cascade in a phytopathogen such as* C*.* higginsianum*, unlike in* S*.* cerevisiae*.

Overall, as a pleiotropic regulator of morphogenesis and plant infection, Ste7 MEK has highly conserved roles in phytopathogens, even those that are phylogenetically diverse. Moreover, there are some functional differences of this gene in several fungal species, which suggest that the regulation of Ste7 MEK varies and may be related to genetic distances between organisms.

## 5. Conclusions

It can be concluded that the* C*.* higginsianum* gene ChSte7 is involved in regulation of vegetative growth, appressorial formation, and postinvasive growth in host tissues. This is an important and conserved virulence factor affecting the infection of* C*.* higginsianum* on cruciferous plants.

## Figures and Tables

**Table 1:** Strains used in this study.

Strain	Description	Reference
Ch-1	*Colletotrichum higginsianum* IMI349063	[4]
Δ*ChSte7-26*	ChSte7 disruption mutant from Ch-1	This study
Δ*ChSte7-48*	ChSte7 disruption mutant from Ch-1	This study
CΔ*ChSte7-26-20*	ChSte7 complementation strain from Δ*ChSte7-26*	This study
EHA105	*Agrobacterium tumefaciens* competent cell	[8]
DH5*α*	*Escherichia coli* competent cell	[8]

**Table 2:** Primers used in this study.

Primer	Sequence (5′ to 3′)
ChSte7FP	ATGGCCGATCCTTTTGCGC
ChSte7RP	CTAGTTGGAGGTACCATTGTACACATCG
ChSte7F1Fp	CCCAAGCTTGGATACTGTCCATCACTTCATCGCC
ChSte7F1Rp	ACGCGTCGACCCATGCATGATCTGGAGCTCACG
ChSte7F2Fp	GCTCTAGAAAAGTGATGAACGCTTGGGCA
ChSte7F2Rp	GGGGTACCGACCCTCCCGTCTCCTTCG
ChSte7-KF	TCGGGTAAGTTTGGCTTTGTTTCA
ChSte7-KR	CTAGTTGGAGGTACCATTGTACACATCG
HphSp	TTCTGCGGGCGATTTGTG
HphAp	AGCGTCTCCGACCTGATG
ChSte7comFP	CCCAAGCTTGCTTAACCATCGGCAACTTCATG
ChSte7comRP	CCCAAGCTTTAGGATAGGAGGCCCTTCCCTGACT
TubulinS	AGAAAGCCTTGCGACGGAACA
TubulinA	CCTCCAGGGTTTCCAGATTA
qRT-STE7F	CAAGAAAGAGATGCGTAAG
qRT-STE7R	GCCGTAGAAGTTGACAATA
qRT-tubulinF	ATGCAGATGTCGTAGAGA
qRT-tubulinR	ACTGTTGTTGAGCCTTAC

Restriction enzyme cutting sites are underlined.

**Table 3:** Growth rate, biomass, conidiation, and conidial germination of the ChSte7 disruption mutants and complementation strain of *C. higginsianum*.

Strain	Growth rate (mm/d)	Mycelial dry weight (mg/mL)	Conidiation (10^6^/plate)	Germination rate (%)
Ch-1	4.3 ± 0.2^a^	2.9 ± 0.1^a^	28.0 ± 2.0^a^	75.5 ± 6.9^a^
Δ*ChSte7-26*	2.4 ± 0.1^b^	1.1 ± 0.2^b^	26.7 ± 1.5^a^	6.2 ± 0.6^b^
Δ*ChSte7-48*	2.5 ± 0.1^b^	0.9 ± 0.2^b^	27.0 ± 0.7^a^	7.5 ± 2.0^b^
CΔ*ChSte7-26-20*	4.2 ± 0.1^a^	2.9 ± 0.2^a^	24.7 ± 0.6^a^	76.5 ± 3.3^a^

Numbers indicated by the same letter in the same column are not significantly different at *P* = 0.05 in a test of LSD.
